# Association of Retinal Vascular Caliber and Age-Related Macular Degeneration in Patients With the Acquired Immunodeficiency Syndrome

**DOI:** 10.1167/iovs.17-23334

**Published:** 2018-02

**Authors:** Douglas A. Jabs, Mark L. Van Natta, Jeong Won Pak, Ronald P. Danis, Peter W. Hunt

**Affiliations:** 1 Department of Ophthalmology, The Icahn School of Medicine at Mount Sinai, New York, New York, United States; 2 Department of Medicine, The Icahn School of Medicine at Mount Sinai, New York, New York, United States; 3 Department of Epidemiology, The Johns Hopkins University Bloomberg School of Public Health, Baltimore, Maryland, United States; 4 Department of Ophthalmology, The University of Wisconsin School of Medicine and Public Health, Madison, Wisconsin, United States; 5 The Department of Medicine, The University of California, San Francisco School of Medicine, San Francisco, California, United States

**Keywords:** acquired immunodeficiency syndrome, age-related macular degeneration, retinal vascular caliber

## Abstract

**Purpose:**

To evaluate the relationship between retinal vascular caliber and AMD in patients with AIDS.

**Methods:**

Participants enrolled in the Longitudinal Study of the Ocular Complications of AIDS had retinal photographs taken at enrollment. Retinal vascular caliber (central retinal artery equivalent [CRAE] and central retinal vein equivalent [CRVE]) and intermediate-stage AMD were determined from these retinal photographs. Photographs were evaluated by graders at a centralized reading center, using the Age-Related Eye Disease Study grading system for AMD and semiautomated techniques for evaluating retinal vascular caliber.

**Results:**

Of the 1171 participants evaluated, 110 (9.4%) had AMD and 1061 (90.6%) did not. Compared with participants without AMD, participants with AMD had larger mean CRAEs (151 ± 16 μm versus 147 ± 16 μm; *P* = 0.009) and mean CRVEs (228 ± 24 μm versus 223 ± 25 μm; *P* = 0.02). The unadjusted differences were: CRAE, 4.3 μm (95% confidence interval [CI] 1.1–7.5; *P* = 0.009) and CRVE, 5.5 μm (95% CI 0.7–10.3; *P* = 0.02). After adjustment for age, race/ethnicity, sex, human immunodeficiency syndrome (HIV) transmission category, smoking, enrollment and nadir CD4^+^ T cells, and enrollment and maximum HIV load, the differences between patients with and without AMD were as follows: CRAE, 5.4 μm (95% CI 2.3–8.5; *P* = 0.001) and CRVE, 6.0 μm (95% CI 1.4–10.6; *P* = 0.01).

**Conclusions:**

In patients with AIDS, AMD is associated with greater retinal arteriolar and venular calibers, suggesting a role for shared pathogenic mechanisms, such as persistent systemic inflammation.

AMD is a leading cause of visual impairment and blindness in persons older than 65 years.^1,2^ Although there are regional differences in the prevalence of AMD, globally, it is estimated that the number of persons with AMD will be 196 million in 2020 and 288 million by 2040.^3^ AMD typically is staged as early, intermediate, or late. Early-stage AMD consists of small drusen. Intermediate-stage AMD consists of extensive medium-size drusen or any large drusen, with or without pigment changes. Late-stage AMD is defined by the presence of either choroidal neovascularization or geographic atrophy.^4–7^ Antiretroviral-treated, immune-restored, human immunodeficiency virus (HIV)-infected persons have a marked reduction in the incidence of opportunistic infections and a substantially increased life span compared with those from the era before modern combination antiretroviral therapy (cART).^8–11^ Despite these improvements, antiretroviral-treated, immune-restored, HIV-infected persons have a substantially shortened life span compared with similarly aged, HIV-uninfected peers, which largely is due to an increased risk of non-AIDS diseases associated with aging (e.g., cardiovascular disease, non-AIDS cancers, metabolic diseases, and neurocognitive decline).^12–16^ This increased risk of age-related diseases suggests that antiretroviral-treated, immune-restored, HIV infection is associated with an “accelerated and/or accentuated aging” phenotype.^12,16^ Consistent with this accentuated aging, patients with AIDS have an approximately 4-fold increased age- and sex-adjusted prevalence of intermediate-stage AMD and an approximately 1.75-fold increased race- and sex-adjusted incidence of intermediate-stage AMD when compared with that in HIV-uninfected cohorts.^17,18^ Patients with AIDS also have retinal vascular calibers consistent with those of HIV-uninfected persons who are on average 10 years older, again consistent with an accelerated/accentuated aging phenotype.^19^ Therefore, we investigated the relationship between AMD and retinal vascular caliber in persons with AIDS enrolled in the Longitudinal Study of the Ocular Complications of AIDS (LSOCA).

## Methods

LSOCA was a prospective, observational, cohort study of patients with AIDS in the era of modern cART.^20,21^ Enrollment began on September 1, 1998, and was completed on July 31, 2011. All participants had AIDS diagnosed according to the 1993 Centers for Disease Control and Prevention revised criteria for the diagnosis of AIDS.^22^ Recruitment occurred at 19 clinical centers throughout the United States, typically located in urban centers with a large HIV-infected population.^20^ Participants with and without ocular opportunistic infections were recruited.

At enrollment, all participants gave a detailed medical and HIV-related disease history; relevant findings were confirmed from the medical record. Fifty to 60-degree retinal photograph of the disc and macula were taken at enrollment, as previously described.^17–21^ Laboratory testing at enrollment included hematology and blood chemistry, blood CD4^+^ T cells, the amount of circulating HIV RNA in the blood (HIV load), and the presence of antibodies to hepatitis C.^20,21^

Approval for the study and its procedures was obtained from the institutional review boards of the individual participating clinical centers and the three resource centers (chairman's office, coordinating center, and reading center). Written, informed consent was obtained from all participants. The study was conducted in accordance with the principles of the Declaration of Helsinki.

### Grading of Retinal Photographs

Photographs were graded at the Studies of the Ocular Complications of AIDS (SOCA) Reading Center at the University of Wisconsin from stereoscopic color photographs of the macula obtained at enrollment. Photographers and camera systems were certified for SOCA photographic procedures by reading center personnel. Images were graded either from 35-mm film, mounted in typical slide mounts and viewed on a light box with a Donaldson 5X stereoscopic viewer, or from digital images displayed on calibrated computer monitors and viewed with a stereoscopic viewer (PS Manufacturing, Portland, OR, USA). The Early Treatment Diabetic Retinopathy Grid, measuring 7.2 mm on the retina, was placed on film slides using an acetate overlay sized for the camera type and degree of view.^5,17,18^ Digital image grading used software tools to calibrate and locate the grid.^7,17,18^ Approximately 10% of the enrollment photographs were digital. Grading of digital images at a reading center for AMD has been demonstrated to be comparable with that of film images.^23^ Fundus photographs were graded for the features of intermediate-stage AMD, including the presence, size, and area of drusen, and the presence and area of pigmentary abnormalities. Grading procedures were based on the Age-Related Eye Disease Study system for classifying AMD from retinal photographs.^6,7^ Graders were masked as to clinical information. The outcome of interest was the presence of intermediate-stage AMD, defined as at least one large druse (>150 μm using modern estimates of the average disc size or >125 μm using the traditional estimate) or extensive medium-sized drusen with pigment abnormalities.^6,24^ Quality control was provided by a resampling and regrading of 10% of the photographs by the reading center project ophthalmologist (RPD).

Retinal vascular indices were determined in a semiautomated manner as previously described.^19,25^ Briefly, the six largest arterioles and venules in a ring-shaped area located between 0.5 and 1.0 disc diameter from the optic margin were identified. Computer software measured the caliber of these individual vessels, then combined them into two summary variables for the eye: the projected caliber sizes of the central retinal artery equivalent (CRAE) and of the central retinal vein equivalent (CRVE). Because drusen are difficult to evaluate in the face of extensive retinal necrosis and scarring from cytomegalovirus (CMV) retinitis, eyes with ocular opportunistic infections were not graded, and these participants were not included in the AMD study.^26^

### Statistical Methods

The association of the presence of AMD at baseline with characteristics of the study population was assessed using the χ^2^ test for categorical variables, the *t*-test for normally distributed continuous variables, and the Wilcoxon rank sum test for non-normally distributed continuous variables.^27^ Multiple linear regression was used to assess relationships of CRAE and CRVE with presence of AMD in three models: unadjusted, adjusted for age, and adjusted for age and other baseline covariates. *P* values were two-sided and nominal. Statistical analyses were conducted with SAS/STAT, version 9.3 (SAS Institute, Inc., Cary, NC, USA) and Stata version 15.0 (StataCorp, College Station, TX, USA) software packages.

## Results

### Characteristics of the Study Population

Of the 2392 participants enrolled in LSOCA, 535 had an intraocular opportunistic infection (primarily CMV retinitis) and could not be assessed for AMD, leaving 1857 participants. Of these, 1583 were assessed for AMD from the enrollment photographs. Of these 1583 participants, 1171 also were assessed for the retinal vascular caliber from enrollment photographs. The 1171 participants assessed for both AMD and retinal vascular caliber form the study population. Of these 1171 patients, 110 (9.4%) had intermediate-stage AMD, and 1061 (90.6%) did not.

Baseline (enrollment) characteristics of the study population and of participants with and without AMD are listed in Table 1. There were no significant differences between participants with and without AMD with respect to sex, race/ethnicity, time since AIDS diagnosis, enrollment CD4^+^ T cells, nadir CD4^+^ T cells before enrollment, HIV load at enrollment, maximum HIV load before enrollment, use of cART, and comorbidities. Participants with AMD were older (mean age 47.0 ± 8.3 years) than those without AMD (mean age 42.6 ± 8.0 years, *P* < 0.0001) and appeared to have a greater proportion of participants whose HIV transmission category was either injection drug use or heterosexual transmission (*P* = 0.04; see Table 1).

**Table 1:**
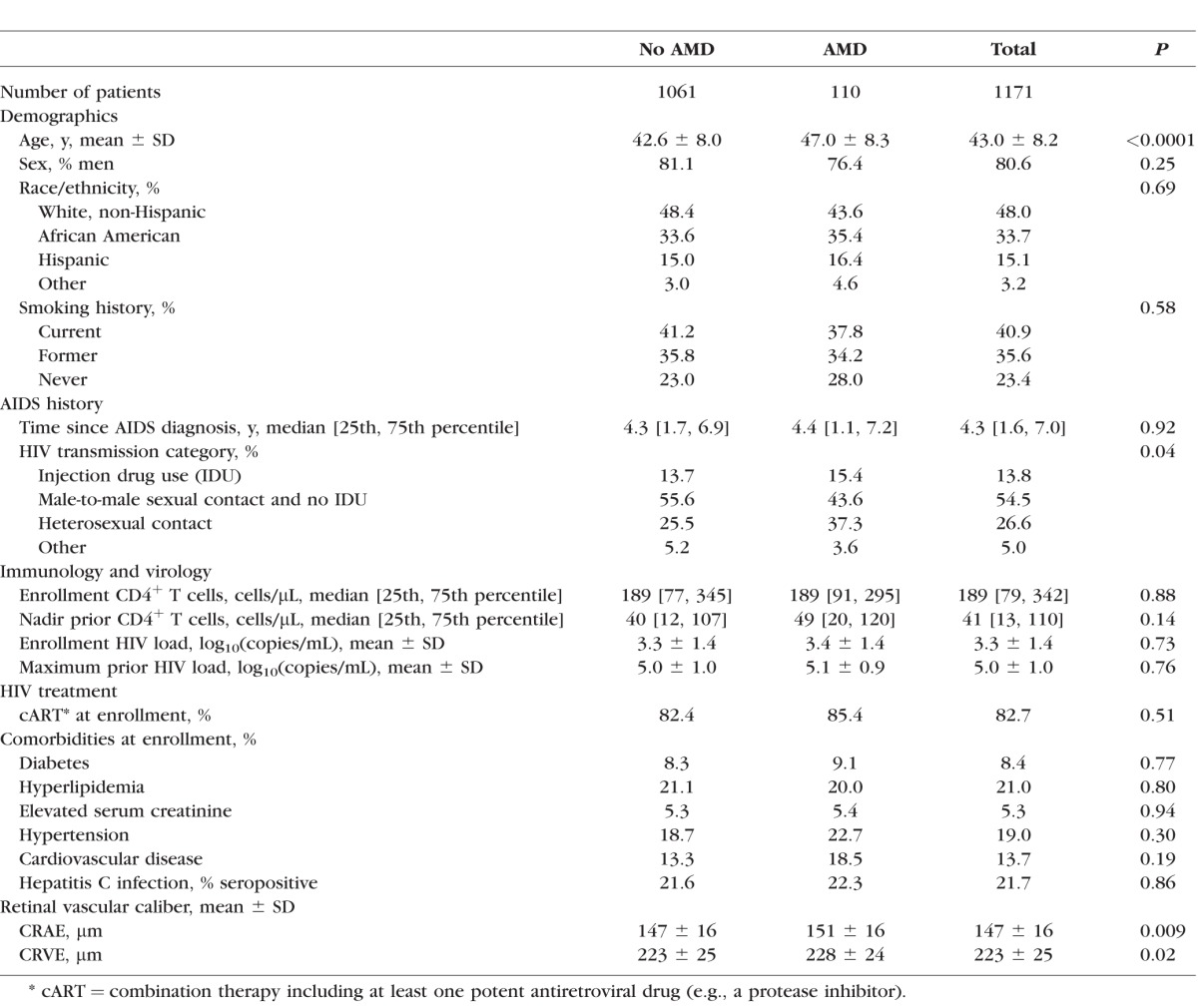
Univariate Analysis of Baseline Patient Characteristics and Prevalent Intermediate-stage AMD in Patients With AIDS Enrolled in LSOCA

### Retinal Vascular Caliber

**Figure 1:**
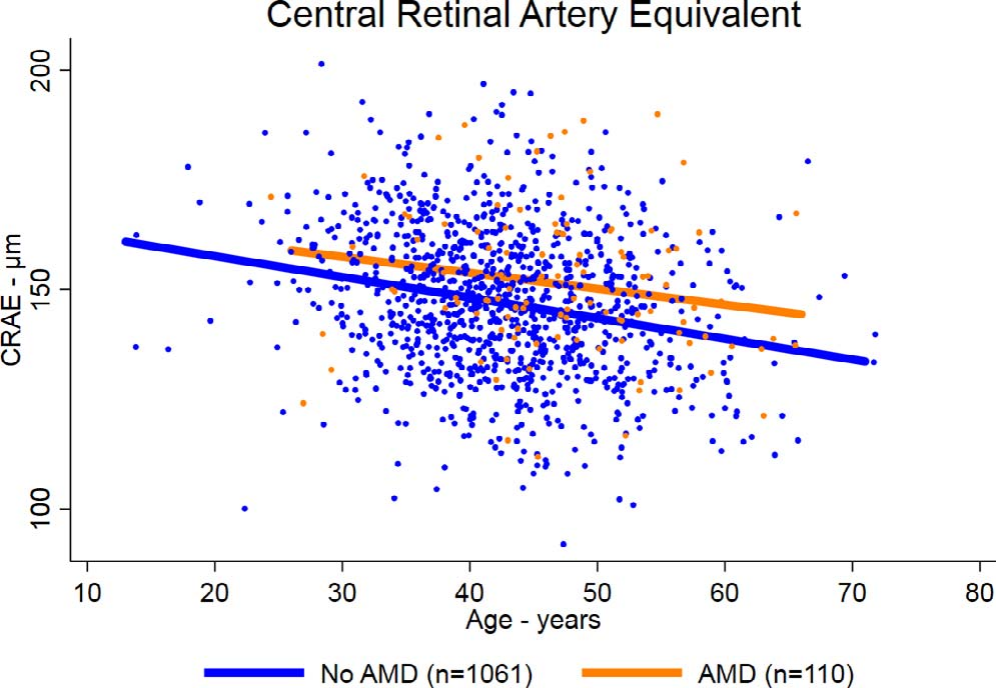
Scatterplot and regression lines of CRAE by age in patients with AIDS with and without AMD.

**Figure 2:**
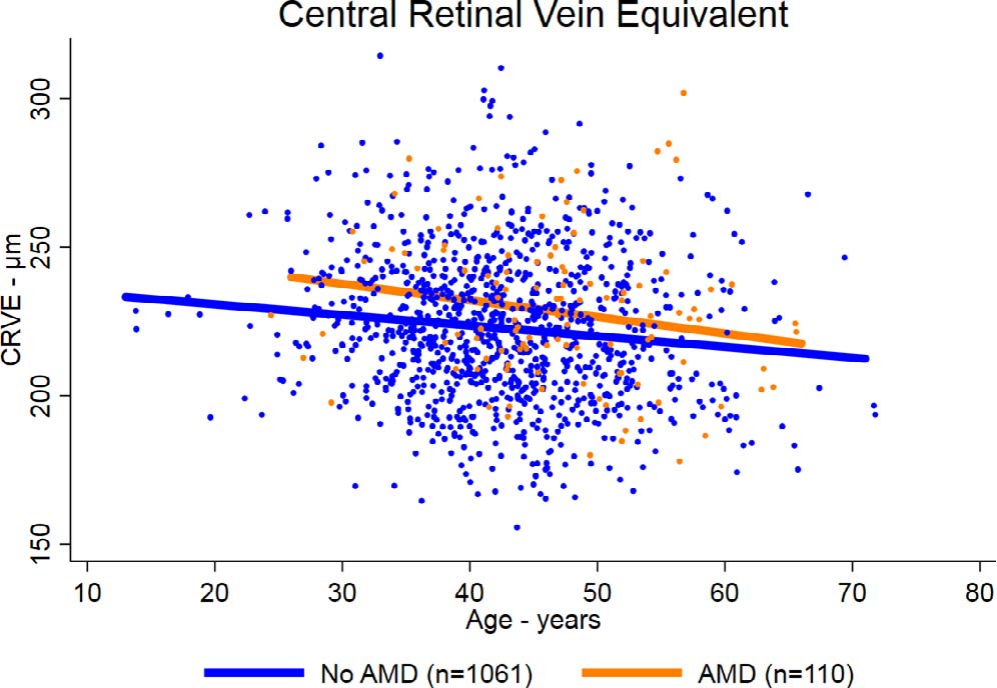
Scatterplot and regression lines of CRVE by age in patients with AIDS with and without AMD.

Participants with AMD had larger CRAE and CRVE than participants without AMD (Table 1; Figs. 1, 2). The mean (± SD) CRAEs for participants with and without AMD were 151 ± 16 μm and 147 ± 16 μm, respectively (*P* < 0.009). The mean (± SD CRVEs for participants with and without AMD were 228 ± 24 μm and 223 ± 25 μm, respectively (*P* = 0.02). The mean CRAE and CRVE declined with age for participants with and without AMD, but were consistently larger for participants with AMD (Figs. 1, 2, respectively). The adjusted differences in CRAE and CRVE between those with and without AMD were even greater in the multiple linear regression analyses (Table 2). In the model adjusted for age, race/ethnicity, sex, HIV transmission category, smoking, enrollment and before enrollment nadir CD4^+^ T cells, enrollment and prior maximum HIV load, the difference in mean CRAE between those with and without AMD was 5.4 μm (95% confidence interval [CI] 2.3–8.5; *P* = 0.001), and the difference in mean CRVE between those with and without AMD was 6.0 μm (95% CI 1.4–10.6; *P* = 0.01).

**Table 2:**
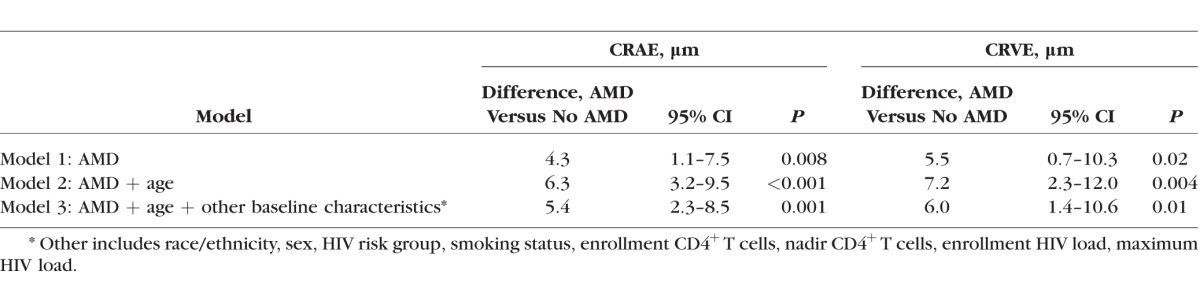
Multivariate Analysis of Baseline Patient Characteristics for Differences in Retinal Vascular Caliber in Patients With AIDS With and Without AMD Enrolled in LSOCA

## Discussion

We previously reported that patients with AIDS had an approximately 4-fold increased prevalence of intermediate-stage AMD and an approximately 1.75-fold increased incidence of intermediate-stage AMD, consistent with the accelerated/accentuated aging phenotype present in antiretroviral-treated, immune-restored, HIV-infected persons.^17,18^ In this study, we demonstrate that persons with AIDS with AMD have larger retinal arteriolar and venular calibers than HIV-infected persons without AMD. Previous work in HIV-uninfected populations has demonstrated several risk factors for changes in retinal vascular caliber.^28–34^ Older age is associated with narrower retinal vascular caliber, and hypertension is associated with narrower retinal arterioles; conversely, cigarette smoking is associated with wider retinal arterioles and venules, although some studies have reported a greater effect on venules.^28–30^ Biomarkers of systemic inflammation consistently are associated with wider retinal venules.^28,29,31,32^ The results of studies of HIV-uninfected persons with AMD have been variable, with one study showing only wider retinal arterioles and another study only wider retinal venules.^28,29,33,34^ In our study, both retinal arteriolar caliber and venular caliber were greater among persons with AMD. This effect was consistent across age, and adjustments for baseline parameters only increased the estimated difference. Our study did show an age-related decline in both CRAE and CRVE in both persons with and without AMD, as is seen in HIV-uninfected persons, but the CRAE and CRVE consistently were larger among patients with AMD than those without.

Antiretroviral-treated, immune-restored, HIV-infected persons have immunologic changes similar to those seen in HIV-uninfected persons older than 70 years, a phenomenon sometimes termed immunosenescence.^12,16^ Antiretroviral-treated, immune-restored, HIV-infected persons are characterized by a state of chronic immune activation with ongoing systemic inflammation.^12,16,35^ Studies in HIV-uninfected persons demonstrate that systemic inflammation is a risk factor for AMD.^36–39^ Therefore, the increased retinal venular caliber in HIV-infected persons with AMD may relate to the common role of systemic inflammation in the pathogenesis of AMD and in altered retinal vascular caliber. Nevertheless, it also is possible that other, unobservable factors are responsible for both the occurrence of AMD and vascular dilation in these patients.

Caution should be taken in interpreting our data. Although the AMD lesions seen in the LSOCA population are clinically and photographically identical to those seen in HIV-uninfected populations, we do not have histology to confirm the nature of the lesions. Caution also should be taken in extrapolating our data to all HIV-infected persons. The LSOCA cohort enrolled only persons with AIDS and not earlier stages of HIV infection.^20,21^ The nadir CD4^+^ T cells were very low, even though many participants had substantial immune recovery by enrollment. The effects of such levels of immune deficiency on subsequent immune activation and systemic inflammation may be different from those seen in HIV-infected patients who never reach this level of immune deficiency. As such, the prevalence and incidence of AMD in HIV-infected patients with earlier stages of HIV infection remain uncertain. Nevertheless, because of late diagnosis and difficulties in completely suppressing HIV replication among all patients, many individuals with HIV infection will present with or progress to AIDS,^40,41^ so that there remains a population of HIV-infected patients who will progress to AIDS and for whom these results are relevant. Furthermore, the LSOCA cohort is similar to the AIDS epidemic in the United States in terms of demographic and other characteristics,^20^ suggesting some degree of generalizability to patients with AIDS. Finally, we only could analyze (and adjust for) those factors where we had data from the LSOCA cohort study. As such, it is possible that other, unobservable factors may contribute to both AMD and retinal vascular dilation in this population.

In sum, LSOCA data demonstrate increased retinal vascular caliber, both CRAE and CRVE, in persons with AIDS and AMD. The relationship of systemic inflammation to AMD in HIV-uninfected persons and to increased retinal venular caliber,^31,32,36–39^ as well as the state of chronic immune activation and systemic inflammation among antiretroviral-treated HIV-infected persons,^12,16,35^ suggest a possible common pathogenetic role for inflammation in both of these findings.

## References

[1] Friedman DS, O'Colmain B, Munoz B, Prevalence of age-related macular degeneration in the United States. *Arch Ophthalmol*. 2004; 122: 564–572. 15078675 10.1001/archopht.122.4.564

[2] Congdon N, O'Colmain B, Klaver CC, Causes and prevalence of visual impairment among adults in the United States. *Arch Ophthalmol*. 2004; 122: 477–485. 15078664 10.1001/archopht.122.4.477

[3] Wang WL, Su X, Cheung CMG, Klein R, Cheng C-Y, Wong TY. Global prevalence of age-related macular degeneration and disease burden projection for 2020 and 2040: a systematic review. *Lancet*. 2014; 2: e106–e116. 10.1016/S2214-109X(13)70145-1 25104651

[4] Davis MD, Gangon RE, Lee LY, The Age-Related Eye Disease Study severity scale for age-related macular degeneration: AREDS report no. 17. *Arch Ophthalmol*. 2005; 123: 1484–1498. 16286610 10.1001/archopht.123.11.1484 PMC1472813

[5] Age-Related Eye Disease Research Group. The Age-Related Eye Disease Study system for classifying age-related macular degeneration from stereoscopic color fundus photographs: Age-Related Eye Study report no. 6. *Am J Ophthalmol*. 2001; 132: 668–681. 11704028 10.1016/s0002-9394(01)01218-1

[6] Ferris FL, Davis MD, Clemons TE, A simplified severity scale for age-related macular degeneration. *Arch Ophthalmol*. 2005; 123: 1570–1574. 16286620 10.1001/archopht.123.11.1570 PMC1473206

[7] Danis RP, Domalpally A, Chew EY, Methods and reproducibility of grading optimized digital color photographs in the Age-Related Eye Disease Study 2 (AREDS 2 report number 2). *Invest Ophthalmol Vis Sci*. 2013; 54: 4548–4554. 23620429 10.1167/iovs.13-11804 PMC3706107

[8] Palella FJ, Delaney KM, Moorman AC, Declining morbidity and mortality among patients with advanced human immunodeficiency virus infection. *N Eng J Med*. 1998; 338: 853–860. 10.1056/NEJM199803263381301 9516219

[9] Holtzer CD, Jacobson MA, Hadley WK, Decline in the rate of specific opportunistic infections at San Francisco General Hospital, 1994–1997. *AIDS*. 1998; 12: 1931–1933. 9792398

[10] Sugar EA, Jabs DA, Ahuja A, Incidence of cytomegalovirus retinitis in the era of highly active antiretroviral therapy. *Am J Ophthalmol*. 2012; 153: 1016–1024. 22310076 10.1016/j.ajo.2011.11.014 PMC3358595

[11] Braithwaite RS, Roberts MS, Chang CC, Influence of alternative thresholds for initiating HIV treatment on quality-adjusted life expectancy. *Ann Intern Med*. 2008; 148: 178–185. 18252681 10.7326/0003-4819-148-3-200802050-00004 PMC3124094

[12] Deeks SG. HIV infection, inflammation, immunosenescence, and aging. *Annual Rev Med*. 2011; 62: 141–155. 21090961 10.1146/annurev-med-042909-093756 PMC3759035

[13] Lohse N, Hansen AB, Pedersen G, Survival of persons with and without HIV infection in Denmark, 1995–2005. *Ann Intern Med*. 2007; 146: 87–95. 17227932 10.7326/0003-4819-146-2-200701160-00003

[14] Hogg R, Lima V, Sterne JA, Life expectancy of individuals on combination antiretroviral therapy in high-income countries: a collaborative analysis of 14 cohort studies. *Lancet*. 2008; 372: 293–299. 18657708 10.1016/S0140-6736(08)61113-7 PMC3130543

[15] Kaplan RC, Kingsley LA, Sarrett AR, Ten-year predicted coronary heart disease risk in HIV-infected men and women. *Clin Infect Dis*. 2007; 45: 1074–1081. 17879928 10.1086/521935

[16] Pathai S, Bajillan H, Landay AL, High KP. Is HIV a model of accelerated or accentuated aging? *J Gerontol A Biol Sci Med Sci*. 2014; 69: 833–842. 24158766 10.1093/gerona/glt168 PMC4067117

[17] Jabs DA, Van Natta ML, Sezgin E, for the Studies of the Ocular Complications of AIDS Research Group Prevalence of intermediate-stage age-related macular degeneration in patients with acquired immunodeficiency syndrome. *Am J Ophthalmol*. 2015; 159: 1115–1122. 25769246 10.1016/j.ajo.2015.01.037 PMC6126535

[18] Jabs DA, Van Natta ML, Pak JW, Danis RP, Hunt PW. Incidence of intermediate-stage age-related macular degeneration in patients with the acquired immunodeficiency syndrome. *Am J Ophthalmol*. 2017; 179: 151–158. 28499708 10.1016/j.ajo.2017.05.004 PMC5523452

[19] Gangaputra S, Kaylani PS, Fawzi AA, for the Studies of the Ocular Complications of AIDS Research Group. Retinal vessel caliber among people with acquired immunodeficiency syndrome: relationships with disease-associated factors and mortality. *Am J Ophthalmol*. 2012; 153: 434–444. 22019225 10.1016/j.ajo.2011.08.028 PMC3289046

[20] Jabs DA, Van Natta ML, Holbrook JT, The Longitudinal Study of the Ocular Complications of AIDS: 1. Ocular diagnoses at enrollment. *Ophthalmology*. 2007; 114: 780–786. 17258320 10.1016/j.ophtha.2006.11.008

[21] Jabs DA, Van Natta ML, Holbrook JT, The Longitudinal Study of the Ocular Complications of AIDS: 2. Ocular examination results at enrollment. *Ophthalmology*. 2007; 114: 787–793. 17210182 10.1016/j.ophtha.2006.07.065

[22] Centers for Disease Control and Prevention. 1993 revised classification system for HIV infection and expanded surveillance case definition for AIDS among adolescents and adults. *Morb Mortal Wkly Rep*. 1992; 41: 1–19. 1361652

[23] Hubbard LD, Danis RP, Neider MW, Brightness, contrast, and color balance of digital versus film retinal images in the Age-Related Eye Disease Study 2. *Invest Ophthalmol Vis Sci*. 2008; 49: 3269–3282. 18421079 10.1167/iovs.07-1267

[24] Ferris FL III, Wilkinson CP, Bird A, on behalf of the Beckman Initiative for Macular Research Classification Committee Clinical classification of age-related macular degeneration. *Ophthalmology*. 2013; 120: 844–851. 23332590 10.1016/j.ophtha.2012.10.036 PMC11551519

[25] Hubbard LD, Brothers RJ, King WN, Methods for evaluation of retinal microvascular abnormalities associated with hypertension/sclerosis in the Atherosclerosis Risk in Communities Study. *Ophthalmology*. 1999; 106: 2269–2280. 10599656 10.1016/s0161-6420(99)90525-0

[26] Jabs DA. Cytomegalovirus retinitis and the acquired immune deficiency syndrome: bench to bedside: LXVII Edward Jackson Memorial Lecture. *Am J Ophthalmol*. 2011; 151: 198–216. 21168815 10.1016/j.ajo.2010.10.018 PMC3057105

[27] Altman DG. Practical Statistics for Medical Research. London: Chapman and Hall; 1991: 213–215, 241–256.

[28] Sun C, Wang JJ, Mackey DA, Wong TY. Retinal vascular caliber: systemic, environmental, and genetic associations. *Surv Ophthalmol*. 2009; 54: 74–95. 19171211 10.1016/j.survophthal.2008.10.003

[29] Ikram MK, Ong YT, Cheung CY, Wong TY. Retinal vascular caliber measurements: clinical significance, current knowledge, and future perspectives. *Ophthalmologica*. 2013; 229: 125–136. 23006932 10.1159/000342158

[30] Liew G, Sharrett R, Wang JJ, Relative importance of systemic determinants of retinal arteriolar and venular caliber: the ARIC Study. *Arch Ophthalmol*. 2008; 126: 1404–1410. 18852419 10.1001/archopht.126.10.1404 PMC2995700

[31] Klein R, Klein BE, Knudson MD, Wong TY, Tsai M. Are inflammatory factors related to retinal vessel caliber? *Arch Ophthalmol*. 2006; 124: 87–94. 16401789 10.1001/archopht.124.1.87

[32] Wong TY, Islam A, Klein R, Retinal vascular caliber, cardiovascular risk factors, and inflammation: the Multi-Ethnic Study of Atherosclerosis (MESA). *Invest Ophthalmol Vis Sci*. 2006; 47: 2341–2350. 16723443 10.1167/iovs.05-1539 PMC2258139

[33] Jeganathan VSE, Kawasaki R, Wang JJ, Retinal vascular caliber and age-related macular degeneration: the Singapore Malay Eye Study. *Am J Ophthalmol*. 2008; 146: 954–959. 18760764 10.1016/j.ajo.2008.07.006

[34] Yang K, Zhan SY, Liang YB, Association of dilated retinal arteriolar caliber with early age-related macular degeneration: the Handan Eye Study. *Graefes Arch Clin Exp Ophthalmol*. 2012; 250: 741–749. 21971892 10.1007/s00417-011-1824-4

[35] Hunt PW, Sinclair E, Rodriguez B, Gut epithelial barrier dysfunction and innate immune activation predict mortality in treated HIV infection. *J Infect Dis*. 2014; 210: 1228–1238. 24755434 10.1093/infdis/jiu238 PMC4192038

[36] Lechanteur YTE, van de Ven JPH, Samilhodzic D, Genetic, behavioral, and sociademographic risk factors for second eye progression in age-related macular degeneration. *Invest Ophthalmol Vis Sci*. 2012; 53: 5846–5852. 22815349 10.1167/iovs.11-7731

[37] Mitta VP, Christen WG, Glynn RJ, C-reactive protein and the incidence of macular degeneration. *JAMA Ophthalmol*. 2013; 131: 507–513. 23392454 10.1001/jamaophthalmol.2013.2303 PMC3625501

[38] Cousins SW, Espinosa-Heidemann DG, Csaky KG. Monocyte activation in patients with age-related macular degeneration: a biomarker of risk for choroidal neovascularization? *Arch Ophthalmol*. 2004; 122: 1013–1018. 15249366 10.1001/archopht.122.7.1013

[39] Nassar K, Grisanti S, Elfar E, Serum cytokines as biomarkers for age-related macular degeneration. *Graefes Arch Clin Exp Ophthalmol*. 2015; 253: 699–704. 25056526 10.1007/s00417-014-2738-8

[40] Shouse RL, Kajese T, Hall HI, Valleroy LA. Late HIV testing–34 states, 1996–2005. *Morbid Mortal Wkly Rep*. 2009; 58: 661–665. 19553901

[41] Edison L, Hughes D, Drenzek C, Kelly J. Prevalence and indicators of viral suppression among persons diagnosed with HIV infection retained in care–Georgia, 2010. *Morb Mortal Wkly Rep*. 2014; 63: 55–58. PMC5779431 24452133

